# Trace Mineral Imbalances in Global Health: Challenges, Biomarkers, and the Role of Serum Analysis

**DOI:** 10.3390/nu17132241

**Published:** 2025-07-07

**Authors:** Marta López-Alonso, Inés Rivas, Marta Miranda

**Affiliations:** 1 Departamento de Patoloxía Animal, Facultade de Veterinaria, Campus Terra, Universidade de Santiago de Compostela, 27002 Lugo, Spain; 2 Escola Universitaria de Enfermería de Lugo, Servizo Galego de Saúde, Universidade de Santiago de Compostela, 27002 Lugo, Spain; i.rivas.fernandez@usc.es; 3 Hospital Universitario Lucus Augusti, 27003 Lugo, Spain; 4 Departamento de Anatomía, Produción Animal e Ciencias Clínicas Veterinarias, Facultade de Veterinaria, Campus Terra, Universidade de Santiago de Compostela, 27002 Lugo, Spain; marta.miranda@usc.es

**Keywords:** essential trace and toxic elements, serum analysis, mineral nutrition, public health, ICP-MS

## Abstract

**Background/Objectives**: Trace minerals (TMs), both essential and toxic, are integral to human physiology, participating in enzymatic reactions, oxidative balance, immune function, and the modulation of chronic disease risk. Despite their importance, imbalances due to deficiencies or toxic exposures are widespread globally. While low-income countries often face overt deficiencies and environmental contamination, middle- and high-income populations increasingly deal with subclinical deficits and chronic toxic metal exposure. This review aims to explore the relevance of serum as a matrix for evaluating TM status across diverse clinical and epidemiological, geographic, and demographic settings. **Methods**: A narrative literature review was conducted focusing on the physiological roles, health impacts, and current biomarker approaches for key essential (e.g., zinc, copper, selenium) and toxic (e.g., lead, mercury, cadmium, arsenic) trace elements. Particular emphasis was placed on studies utilizing serum analysis and on recent advances in multi-element detection using inductively coupled plasma mass spectrometry (ICP-MS). **Results**: Serum was identified as a versatile and informative matrix for TM assessment, offering advantages in terms of clinical accessibility, biomarker reliability, and capacity for the simultaneous quantification of multiple elements. For essential TMs, serum levels reflect nutritional status with reasonable accuracy. For toxic elements, detection depends on instrument sensitivity, but serum can still provide valuable exposure data. The method’s scalability supports applications ranging from public health surveillance to individualized patient care. **Conclusions**: Serum trace mineral analysis is a practical and scalable approach for nutritional assessment and exposure monitoring. Integrating it into clinical practice and public health strategies can improve the early detection of imbalances, guide interventions such as nutritional supplementation, dietary modifications, and exposure mitigation efforts. This approach also supports advanced personalized nutrition and preventive care.

## 1. Introduction

Trace minerals (TMs) are essential in numerous metabolic functions, playing a pivotal role in human health [1]. TMs facilitate critical processes including enzyme activation, immune system support, and protection against oxidative stress, all of which are vital for maintaining overall physiological well-being. Traditionally, TM deficiency associated with malnutrition was the main type of problem observed, particularly in low-income countries, due to limited access to mineral-rich foods. However, recent studies have expanded our understanding, highlighting the importance of TM imbalances (both low and high levels) in high-income countries, particularly in relation to chronic diseases and aging [2]. Lifestyle choices, such as adopting vegan and/or high-fiber diets, and the aging demographic can lead to TM imbalances, particularly in subclinical processes [3]. Such imbalances are linked to the development of a wide range of chronic diseases (including cardiovascular conditions, neurodegenerative disorders, and cancers) and also to an exacerbation of these [4]. Moreover, managing TM imbalances can have therapeutic benefits, helping to mitigate the effects of these diseases. Despite the essential involvement of TMs in healthy aging, controlling inflammation, and reducing oxidative stress, data on individual mineral status have traditionally been scarce in the human population.

Traditionally, the assessment of TM status at the population level has been conducted indirectly through estimations of dietary intake, based on food consumption and the tabulated data of TM concentrations in food [5]. These methods have been very useful for making general evaluations and addressing the problem of TM deficiencies in low-income countries, where the main limitation is the lack of food. However, they cannot capture the individual variability that is particularly evident in high-income societies. This variability, largely due to lifestyle choices and an aging population, greatly influences the bioavailability of TMs and physiological needs. Addressing this complex situation is essential, as it will enable the development of more precisely targeted, effective health interventions adjusted to meet individual requirements, particularly in regions with advanced healthcare systems [4].

Advancements in TM analysis methods, including multi-element techniques with very low detection limits, have led to unprecedented levels of precision, overcoming challenges previously considered insurmountable [6]. Such advancements enable the routine determination of TM status in populations directly from biological samples, particularly serum. Used together with powerful statistical tools and artificial intelligence, these methods can enhance our understanding of how lifestyle and nutritional factors influence individual health outcomes, thereby facilitating personalized and population-level interventions [7]. 

This review addresses the urgent global challenges in TM nutrition that impact diverse populations, ranging from those in areas with limited food supplies to affluent societies influenced by lifestyle choices. Highlighting the role of TMs in the pathogenesis of chronic diseases, we advocate for a paradigm shift towards personalized TM profile analysis. Serum analysis provides a direct and physiologically meaningful measure of TM status. Compared to other biomarker approaches—such as enzyme activity, hormone levels, or urinary excretion—serum concentrations more accurately reflect systemic availability and are less influenced by transient factors such as inflammation, recent intake, or homeostatic regulation. By moving from conventional dietary estimations to precise, direct assessments at the individual level, we aim to harness the precision of advanced serum TM analytical methodologies. These techniques could revolutionize risk stratification and the development of customized therapeutic strategies, significantly impacting personalized healthcare and global public health frameworks.

In addition to essential TMs, this review also considers the impacts of the most important toxic elements, i.e., arsenic (As), cadmium (Cd), lead (Pb), and mercury (Hg), which pose health risks both by themselves and through their interactions with essential elements. These toxic elements can enter the human body through contaminated food sources, industrial exposure, and environmental pollution. Their presence can interfere with metabolic pathways and detoxification processes, disrupting the balance of essential nutrients and exacerbating health issues. These interactions highlight the need for a detailed understanding of both essential and toxic TMs to design effective health interventions and preventive measures. This comprehensive approach allows us to address the broad spectrum of TM dynamics that contribute to health and disease, reinforcing the need for precise serum analysis in public health strategies.

## 2. Essential Functions of Trace Minerals

Although required in only minute amounts, essential trace elements for humans include chromium (Cr), copper (Cu), iron (Fe), iodine (I), manganese (Mn), molybdenum (Mo), selenium (Se), and zinc (Zn). These elements are indispensable for maintaining physiological homeostasis and play distinct yet interconnected critical roles across a wide array of cellular and systemic processes essential for health (Table 1). Some of these elements contribute to structural integrity; e.g., Zn is involved in gene regulation through Zn-finger proteins, and Cu is involved in connective tissue formation. Many elements act as enzymatic cofactors; e.g., Zn, Cu, Mn, Mo, and Se support metabolic pathways, redox balance, and cellular signaling. Iodine and Se are key regulators of thyroid hormone metabolism, while Fe facilitates oxygen transport via hemoglobin and myoglobin, and Cu assists in Fe metabolism and erythropoiesis. The immune system depends on Zn, Se, and Cu for correct functioning, as these elements influence immune cell proliferation and oxidative stress control. Additionally, Se, Zn, Cu, and Mn play central roles in antioxidant defense, protecting tissues from damage caused by reactive oxygen species. Chromium enhances insulin sensitivity and glucose metabolism, while Fe, Cu, Zn, and Se are essential for neuroprotection and cognitive function [4]. Maintaining tight homeostatic control of these trace elements is crucial, as even slight imbalances can disrupt cellular processes and increase the risk of disease.

The human body regulates TM levels through specific mechanisms such as intestinal absorption (e.g., DMT1 for iron) and renal excretion (e.g., urinary clearance of iodine), which help maintain physiological homeostasis and prevent toxicity. Most TMs, including Zn, Cu, Fe, Mn, and Cr, are primarily regulated at the intestinal level, where absorption is modulated in response to physiological demand. Specific transporters, such as divalent metal transporter 1 (DMT1) (for Fe) and metallothioneins (for Zn and Cu), tightly control uptake and limit excessive accumulation. By contrast, Se, I, and Mo are primarily regulated at the renal level, where excretion plays a key role in maintaining systemic balance. For example, Se is excreted in urine as various Se compounds, playing a key role in its systemic balance, while I homeostasis is maintained through renal clearance, reflecting thyroid hormone demands. The liver also has a critical role in storing and redistributing trace elements, particularly Cu and Fe, which are transported via ceruloplasmin and transferrin, respectively [4].

**Figure 1:**
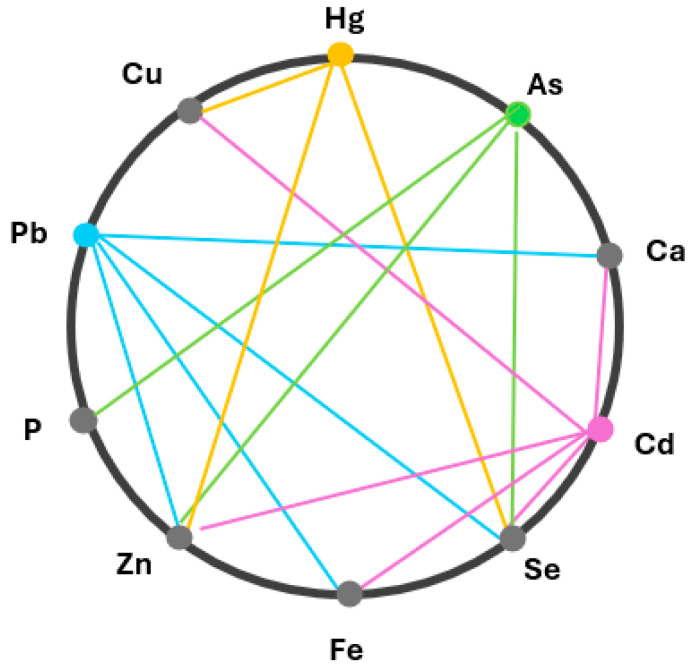
Schematic representation of experimentally supported interactions between toxic and essential trace elements. Lines indicate known interferences, such as competition for absorption, enzymatic inhibition, or displacement. Based on evidence from [16].

Finally, it is important to consider how toxic elements can interfere with the vital functions of TM. Numerous interactions between toxic and essential elements have been experimentally documented, affecting absorption, transport, enzyme activity, and physiological balance. According to the World Health Organization, such interactions are particularly well-established for Cd, Pb, Hg, and As, each of which can disrupt the homeostasis of multiple essential minerals [16]. Figure 1 provides a visual summary of the most relevant toxic–essential relationships. For instance, Cd can inhibit the activity of Zn-dependent enzymes, disrupting essential biochemical pathways that are critical for immune and reproductive system health [17]. Mercury binds to Se, preventing it from acting as an antioxidant in various enzymes, which compromises cellular defense against oxidative stress [18]. Arsenic competes with phosphate groups, interfering with cellular processes reliant on phosphorylation for signal transduction and energy metabolism [19]. Additionally, Pb can displace Ca (a macroelement) in bone, affecting bone health and structural integrity [20]. Such interactions highlight the complex dynamics between toxic and essential TMs, necessitating careful balance to prevent detrimental effects on health.

## 3. Essential Trace Mineral Imbalances (Deficiencies and Excesses)

Maintaining adequate TM levels is crucial for physiological balance. For instance, zinc deficiency impairs immune function and wound healing, while excess copper can exacerbate oxidative stress and promote angiogenesis, contributing to disease progression. Although the body regulates mineral levels through various mechanisms, external and internal factors such as inadequate dietary intake, genetic conditions, malabsorption disorders, chronic diseases, and environmental exposure can lead to imbalances. The severity and clinical manifestations of these disturbances vary depending on the specific mineral considered, as well as the life stage and overall health status of the individual. Certain populations, including infants, pregnant women, and the elderly, are particularly vulnerable to deficiencies due to increased physiological demands, while excessive intake or impaired excretion can lead to toxic effects. This section provides an overview of the health consequences of TM imbalances and discusses the most important causes and effects. While not intended as a comprehensive review of TM deficiency–toxicity, it highlights those aspects that are most relevant and impactful for the general population. For detailed information on TM requirements, deficiencies, and toxicities, readers can consult the recent paper by Ross and coworkers [21], which provides extensive evaluations on these topics.

### 3.1. Iron

Iron deficiency is the most prevalent micronutrient deficiency worldwide, primarily affecting women of reproductive age, infants, and individuals with chronic diseases. According to the World Health Organization, anemia affects over 30% of women of reproductive age and is primarily attributed to iron deficiency in most regions [22]. The most common cause is insufficient dietary intake, particularly in populations with a low consumption of hem Fe from animal sources. Increased Fe demand during pregnancy, menstruation-related blood loss in premenopausal women, and chronic gastrointestinal bleeding, as in inflammatory bowel disease (IBD) or *Helicobacter pylori* infection, also contribute to Fe deficiency [23]. The risk is further exacerbated by malabsorption syndromes, as observed in celiac disease and after bariatric surgery [24]. Additionally, Pb competes with Fe for absorption by binding to the DMT1 in the intestinal mucosa, which can exacerbate Fe deficiency, particularly in vulnerable populations such as children and pregnant women [25]. Iron deficiency leads to Fe-deficiency anemia, characterized by fatigue, weakness, cognitive impairment, and increased susceptibility to infections due to reduced oxygen transport. In children, it can impair neurodevelopment and learning capacity [26], while in pregnant women, it increases the risk of preterm birth and low birth weight [27].

Conversely, Fe overload, often resulting from genetic disorders, such as hereditary hemochromatosis, excessive supplementation, or repeated blood transfusions, is associated with oxidative stress and inflammation [28]. Excess Fe accumulates in tissues, particularly in the liver, heart, and pancreas, increasing the risk of cirrhosis, cardiomyopathy, and diabetes. The redox activity of iron, particularly its capacity to cycle between the Fe^2+^ (ferrous) and Fe^3+^ (ferric) states, plays a central role in oxidative stress. Through Fenton chemistry, ferrous iron catalyzes the generation of reactive oxygen species (ROS), contributing to cellular damage when iron accumulates beyond safe thresholds. Additionally, Fe deposition in the brain has been implicated in neurodegenerative diseases such as Alzheimer’s and Parkinson’s, where it exacerbates neuronal death via oxidative stress [29,30].

### 3.2. Zinc

Zinc deficiency affects approximately one-third of the global population, with the highest burden in low-income countries where dietary intake is insufficient [31]. It is commonly associated with malabsorption syndromes, including IBD, chronic diarrhea, and celiac disease, as well as prolonged parenteral nutrition without adequate supplementation [32]. Chronic alcohol consumption and bariatric surgery can also impair Zn absorption. Additionally, environmental exposure to Cd can further exacerbate Zn deficiency by competing for binding sites on metallothionein and other Zn transporters, reducing its bioavailability and aggravating deficiency symptoms [33]. Zinc deficiency leads to immunosuppression, delayed wound healing, and increased susceptibility to infections such as pneumonia and diarrhea. In children, inadequate Zn levels contribute to growth retardation and cognitive deficits, while in adults, they are linked to dermatitis, reproductive issues, and delayed recovery from illness [34,35]. In the elderly, low Zn levels exacerbate age-related macular degeneration and neurodegeneration [36].

Excess Zn, although less common, can lead to Cu deficiency due to the competitive inhibition of Cu absorption, resulting in anemia, neutropenia, and immune dysfunction [37]. Prolonged excessive intake may also impair gastrointestinal function and lead to nausea and vomiting. 

### 3.3. Iodine

Iodine deficiency remains a significant public health concern, particularly in regions with I-deficient soil, affecting nearly two billion people worldwide [38]. It is exacerbated by a low dietary intake of seafood and dairy products, which are primary I sources. Iodine is essential for thyroid hormone synthesis, and deficiency leads to hypothyroidism, goiter, and impaired metabolic regulation. In pregnant women, I deficiency increases the risk of cretinism, a condition characterized by severe intellectual disability, stunted growth, and speech and hearing impairments in offspring [39]. Mild-to-moderate I deficiency during pregnancy and early childhood has been associated with a lower intelligence quotient (IQ) and cognitive impairment.

Excessive I intake, often due to over-supplementation or a high consumption of seaweed, can disrupt thyroid function, triggering hyperthyroidism or autoimmune thyroiditis in susceptible individuals [40].

### 3.4. Selenium

Selenium deficiency is particularly prevalent in areas with Se-poor soil, leading to reduced dietary intake. It has been linked to Keshan disease, a form of cardiomyopathy exacerbated by Coxsackievirus, and Kashin–Beck disease, an osteoarthropathy affecting bone and cartilage [41]. Selenium deficiency also weakens immune defense, increases oxidative stress, and contributes to cognitive decline and thyroid dysfunction by impairing deiodinase activity [42]. Additionally, Se deficiency has been associated with cognitive decline, cardiovascular diseases, and reproductive disorders, including infertility in men and pregnancy complications such as preeclampsia [43,44]. Furthermore, environmental exposure to Hg can exacerbate Se deficiency, as Hg binds strongly to Se, forming a complex that deactivates and thus hinders the biological function of Se-dependent antioxidant enzymes [45].

Excess Se intake leads to selenosis, which manifests as brittle nails, alopecia, gastrointestinal distress, and neurological symptoms [46]. Chronic high Se levels have been weakly associated with an increased risk of type 2 diabetes, although further research is needed to confirm this relationship [47]. In addition, recent large-scale trials have questioned the benefits of supranutritional selenium supplementation, particularly in cancer prevention, reinforcing the importance of maintaining Se intake within the optimal physiological range [44].

### 3.5. Copper

Copper deficiency is relatively rare but can occur in individuals with prolonged parenteral nutrition, excessive Zn intake, or who have undergone bariatric surgery [48]. Excessive intake of Zn, for instance, can lead to Cu deficiency due to competitive inhibition at sites of absorption and displacement from its biological binding sites, which significantly impedes the bioavailability and utilization of Cu. Copper deficiency is associated with anemia, neutropenia, and neurological impairments, including myelopathy and peripheral neuropathy. This deficiency contributes to osteoporosis due to the role of Cu in collagen synthesis and bone remodeling [49].

On the other hand, excess Cu poses significant health risks due to its pro-oxidant properties. Elevated Cu levels are associated with increased oxidative stress and angiogenesis, processes that facilitate tumor growth and metastasis [50]. High serum Cu levels are frequently observed in conditions like Wilson’s disease, a genetic disorder characterized by Cu accumulation in tissues, leading to liver damage, neurological symptoms, and psychiatric disturbances [51]. Beyond its role in cancer progression, Cu imbalance has been linked to cardiovascular diseases, as excessive levels can contribute to oxidative damage in the vascular system [52]. These findings emphasize the dual role of Cu as both a necessary nutrient and a contributor to pathological processes when dysregulated.

### 3.6. Chromium

Chromium deficiency is rare but has been linked to impaired glucose tolerance and an increased risk of type 2 diabetes [53]. While trivalent chromium (Cr^3+^) is considered the biologically active form involved in carbohydrate and lipid metabolism, hexavalent chromium (Cr^6+^) is highly toxic. It readily crosses cell membranes and induces oxidative DNA damage, contributing to its mutagenic and carcinogenic potential. Cr^6+^ exposure, particularly in occupational settings, has been associated with an increased lung cancer risk [54]. This dual nature highlights the importance of distinguishing chromium species when evaluating nutritional status or environmental exposure.

### 3.7. Manganese

Manganese deficiency is uncommon but can result from severe malnutrition or impaired intestinal absorption. It is associated with skeletal abnormalities, impaired glucose metabolism, and neurological dysfunction [55]. However, Mn toxicity is of particular concern in workers exposed to Mn dust in mining and welding industries. Chronic Mn overexposure leads to manganism, a neurological disorder with symptoms resembling Parkinson’s disease [56].

### 3.8. Molybdenum

Molybdenum deficiency is extremely rare and is mainly associated with genetic disorders affecting Mo cofactor synthesis, leading to severe neurological dysfunction [57]. Excess Mo intake, although uncommon, may interfere with Cu metabolism, contributing to secondary Cu deficiency [58]. Elevated Mo exposure has been reported in regions with naturally Mo-rich soils and in certain industrial settings, although clinical toxicity remains rare [58].

## 4. Toxic Element Adverse Effects

Toxic elements such as As, Cd, Hg, and Pb are widely present in the environment and can accumulate in the human body, leading to severe health effects. Exposure occurs through contaminated water, food, air, and industrial processes, affecting multiple organ systems, including the nervous, renal, and cardiovascular systems. Given the persistence and toxicity of these elements, understanding their adverse effects is essential for mitigating risks and protecting vulnerable populations. The main mechanisms through which these toxic elements exert their harmful effects—often involving oxidative stress, interference with essential trace elements, and damage to key cellular pathways—are summarized in Table 2.

### 4.1. Arsenic

Arsenic occurs naturally in the earth’s crust, frequently contaminates groundwater, and is present in certain industrial and agricultural emissions. It accumulates in the body after the consumption of contaminated water and food, particularly of products from areas with high natural deposits [59]. Arsenic exists in both organic and inorganic forms, with the inorganic form being particularly hazardous due to its potent toxicity and carcinogenic properties [60]. The primary dietary sources of As include contaminated groundwater, rice, and seafood, which can contain higher levels of the less toxic organic As. Chronic exposure to inorganic As is linked to an increased risk of cancers such as skin, lung, bladder, and kidney cancers. In addition, As exposure can lead to dermatological changes, including alterations in skin pigmentation and hyperkeratosis, which may appear after years of exposure and serve as early indicators of toxicity. Furthermore, the prolonged ingestion of As-contaminated water has been associated with neurodevelopmental impairments in children, increased susceptibility to infections, and an elevated risk of hypertension. Given the severe health risks associated with As exposure, stringent control regulations and continuous environmental monitoring are essential, especially in regions with naturally high levels of this element. 

### 4.2. Cadmium

Cadmium is a toxic heavy metal present in industrial by-products, tobacco smoke, and phosphate fertilizers, with human exposure occurring through the inhalation or ingestion of contaminated foods such as shellfish, liver, and kidney, and of crops grown in Cd-rich soils [61]. Known for its long biological half-life, Cd can accumulate in the human body, particularly in the kidneys and liver, where it can remain for decades, leading to chronic health issues. Significant health risks associated with Cd exposure include kidney damage, which can progress to kidney failure, bone demineralization resulting in osteoporosis, and an increased risk of lung cancer (Cd is classified as a carcinogen) [62]. Cadmium exerts toxic effects even at low exposure levels, and current evidence indicates that no safe threshold has been established, underscoring the need for stringent exposure limits. Public health measures focus on reducing Cd exposure by regulating industrial emissions, promoting the cessation of smoking, and monitoring food products for Cd content to minimize intake and prevent the long-term health consequences associated with this harmful metal.

### 4.3. Lead

Lead is a pervasive environmental contaminant with no known safe level of exposure. Lead was historically used in a variety of products, including paint, petrol, and plumbing materials. Although regulations have led to a significant reduction in the use of Pb, exposure still occurs primarily through the ingestion of contaminated dust, water from aging lead pipes, and certain foods, as well as through the inhalation of Pb particles from residual industrial emissions [63]. The health risks of Pb are profound, particularly for children, who are highly susceptible to its neurotoxic effects [64]. Even low levels of exposure to lead in children can lead to irreversible cognitive impairment, including a reduced IQ, behavioral problems, and learning disabilities, which can adversely affect educational and social development [63]. In adults, chronic exposure to Pb is linked to an increased risk of hypertension, kidney damage, and cardiovascular diseases. Lead also tends to accumulate in the bones, where it can remain for decades, potentially re-entering the bloodstream long after initial exposure has ceased. Even after remediation, Pb can persist in the body for decades, posing long-term health risks. To mitigate these effects, public health efforts focus on eliminating lead-based paint, replacing old water pipes, and enforcing strict industrial regulations [65].

### 4.4. Mercury

Mercury is a highly toxic element that primarily enters the environment through industrial processes, such as coal burning and waste incineration, and is also released naturally from volcanic activity. Human exposure to Hg can occur via the inhalation of Hg vapor in industrial settings, the consumption of contaminated fish and seafood, and the use of products containing Hg [66]. Mercury exists in various chemical forms, each with distinct toxicokinetic properties. Elemental mercury (Hg^0^), inorganic salts (Hg^+^/Hg^2+^), and organic forms like methylmercury (MeHg^+^) differ markedly in absorption, distribution, and toxicity. The most hazardous form of Hg is methylmercury, which is produced by the microbial methylation of inorganic mercury in aquatic systems and accumulates in the food chain, particularly in predatory fish. Methylmercury is easily absorbed by the human body upon ingestion and can cross both the blood–brain and placental barriers, leading to significant neurological and developmental damage [67]. In adults, chronic exposure to Hg can result in tremors, cognitive deficits, and sensory impairments, while prenatal exposure is particularly dangerous, potentially causing birth defects and developmental disorders in children [65]. Recognizing the global nature of Hg pollution, international agreements (such as the Minamata Convention on Mercury) aim to reduce Hg emissions and phase out products containing Hg, reflecting the critical need for coordinated efforts to mitigate the health impacts on populations worldwide. 

## 5. Trace Minerals and Disease Associations

Although TMs are essential components of numerous biological processes, their roles extend beyond direct causality in diseases related to overt deficiencies and excesses. These elements strongly influence disease progression through mechanisms such as the modulation of oxidative stress, the control of inflammation, and the regulation of the immune system and hormonal balance. Ensuring a correct balance of TMs is crucial for both the prevention and management of specific diseases as well as for maintaining general health. By contrast, toxic elements can exert detrimental effects, often exacerbating the same conditions they cause, through enhanced oxidative stress and immune dysregulation.

This section explores how even subclinical variations in TM levels, including low-environmental toxic element exposure, can predispose individuals to major health conditions, such as cardiovascular diseases, cancer, and neurodegenerative disorders, and can even exacerbate these conditions. The interplay between essential and toxic elements highlights the need for comprehensive strategies that optimize protective TMs like Se and Zn, while minimizing exposure to toxic metals. This need highlights the importance of precise monitoring and targeted interventions, particularly for individuals at high risk, and reinforces the potential of essential TM modulation as a strategic component in disease management and therapy. 

### 5.1. Cardiovascular Diseases

Essential TMs play an essential role in cardiovascular health by influencing key physiological processes, including oxidative stress regulation, inflammation control, and endothelial function. Selenium and Zn have antioxidant properties, which help mitigate oxidative damage, a major contributor to atherosclerosis and other cardiovascular diseases (CVDs). Selenium, through its role in selenoproteins such as glutathione peroxidase (GPx), has been associated with a reduced cardiovascular risk by protecting against lipid peroxidation and oxidative damage in vascular tissues [68]. Zinc also exerts protective effects by modulating vascular inflammation and lipid metabolism. Recent meta-analyses suggest that both low and high zinc levels may influence cardiovascular outcomes, highlighting the importance of maintaining an adequate zinc status [69,70]. Copper and Fe are also critical for cardiovascular function but require tight homeostatic control to prevent adverse effects. Copper is involved in maintaining heart muscle integrity and energy production, but elevated circulating levels have been consistently associated with increased cardiovascular and all-cause mortality, as shown in recent meta-analyses [71]. Conversely, Cu deficiency has been linked to impaired myocardial function and weakened vascular integrity [72]. Similarly, although Fe plays a crucial role in oxygen transport and erythropoiesis, Fe overload can catalyze the production of reactive oxygen species, exacerbating inflammation and endothelial damage, which are key contributors to atherosclerosis and heart failure [73].

In addition to imbalances in essential TMs, exposure to toxic elements further exacerbates the risk of CVDs through overlapping mechanisms, including oxidative stress, endothelial dysfunction, and vascular inflammation. For example, Cd interferes with cellular calcium (Ca) homeostasis and promotes vascular dysfunction, while Pb raises blood pressure by disrupting hormonal systems that regulate vasoconstriction and vasodilation [74]. Moreover, Hg and As can directly damage heart cells and blood vessels, accelerating the process of atherosclerosis [75]. The accumulation of these toxic elements in the cardiovascular system increases the risk of hypertension, heart attacks, and strokes and also exacerbates existing CVDs, making it imperative to develop strategies to minimize exposure to these harmful elements as an integral part of managing cardiovascular health.

### 5.2. Diabetes Mellitus

Essential TMs play a critical role in the metabolic regulation and management of diabetes, influencing a range of physiological functions, ranging from insulin sensitivity to glucose metabolism. Chromium is renowned for enhancing the action of insulin, thus improving glucose uptake in type 2 diabetes patients. Recent studies, including those by Dubey and coworkers [76], have confirmed that Cr supplementation can significantly improve blood sugar levels and insulin sensitivity in such patients. Additionally, Jia and coworkers [77] provide evidence that vanadium may mimic the effects of insulin and enhance glucose metabolism, although further research is needed to validate the long-term benefits and safety. Molybdenum has less direct effects but has critical functions in amino acid metabolism and antioxidant defense [78]. Thus, Mo has broad impacts on metabolic health and indirectly affects diabetes management. Magnesium and Zn are particularly critical, with research showing that deficiencies in these TMs are linked to increased oxidative stress and a higher incidence of diabetic complications [79,80]. The interplay between TMs such as Zn and Cu also has significant effects on diabetes management. Excessive Zn intake can impact Cu absorption negatively, which is essential for cardiovascular and nervous system health, which directly influence diabetes management [81]. Moreover, Se levels must be carefully managed to optimize the protection provided by this TM against oxidative stress while avoiding exacerbating diabetes risk, as both low and high Se levels can be detrimental [79]. A recent meta-analysis highlighted that higher selenium status or prolonged supplementation may be associated with an increased risk of type 2 diabetes, particularly in individuals with already adequate Se levels [82]. Exposure to toxic elements has been associated with an increased risk of type 2 diabetes through both shared and distinct mechanisms [83,84]. One key pathway involves oxidative stress and chronic inflammation, which contribute to insulin resistance and pancreatic β-cell dysfunction. Arsenic, Cd, and Hg directly impair mitochondrial function, disrupting cellular energy metabolism and insulin secretion. Cadmium and Pb interfere with Ca homeostasis, affecting insulin release from β-cells, while Pb further exacerbates metabolic dysfunction by disrupting insulin receptor signaling and depleting essential antioxidants such as Zn. Mercury contributes to β-cell toxicity by interfering with Se-dependent antioxidant systems, reducing insulin synthesis, and exacerbating oxidative damage. These mechanisms collectively highlight the role of toxic elements in metabolic disorders, reinforcing the need to limit environmental exposure in order to mitigate diabetes risk.

### 5.3. Cancer

Essential TMs are increasingly recognized for their complex roles in the oncogenesis and progression of cancer. Elements such as Zn, Se, and Cu are critical for regulating DNA repair, cell proliferation, and apoptosis, influencing both cancer development and patient responses to cancer therapies. Zinc is essential for DNA synthesis and repair, both of which have anti-tumor functions by modulating cellular proliferation and apoptosis. Zinc deficiencies have been linked to an increased risk of prostate and colorectal cancers, possibly due to the reduced protection of DNA from oxidative damage [85]. Selenium functions as a powerful antioxidant and is thus critical in preventing cancer by contributing to detoxification and immune regulation. Epidemiological studies have consistently shown a lower incidence of cancers, particularly prostate and lung cancers, in individuals with adequate Se intake, highlighting the protective effects of selenoproteins [41]. Conversely, the role of Cu in angiogenesis and metastasis illustrates how TM imbalances can accelerate cancer progression, necessitating the careful management of Cu levels [86]. The interplay between TMs and cancer is further evidenced by the significant changes in the blood and tumor tissues of cancer patients, in whom typical alterations include elevated Cu levels and decreased Zn concentrations, impacting disease mechanisms and outcomes [87]. Additionally, recent studies highlight the prognostic importance of TMs such as Se, particularly in regions with low soil Se levels, demonstrating that higher serum Se levels are correlated with improved survival rates in breast, lung, and laryngeal cancer patients [88,89]. Beyond their role in cancer progression, essential TMs are pivotal in modulating the effects of oncological treatments. The inclusion of TMs in conventional therapies such as chemotherapy and radiotherapy has shown promise in enhancing treatment efficacy and reducing toxicity. For instance, Se supplementation has been observed to protect normal cells from chemotherapy-induced toxicity and amplify the therapeutic effects of treatment across various types of cancers [90,91]. However, findings from large intervention trials have raised concerns about the efficacy and safety of supranutritional selenium supplementation in cancer prevention, with some studies reporting neutral or even adverse outcomes [44]. Zinc supplementation has also been effective in reducing chemotherapy-induced mucositis and maintaining quality of life in cancer patients undergoing treatment [92,93]. Overall, the current understanding of TMs in cancer—including roles in prevention, progression, treatment, and prognosis—highlights the need for a detailed, individualized approach to managing TM levels in oncological care. Further research is crucial to better define these roles and optimize TM supplementation strategies, ultimately improving treatment efficacy and enhancing patient quality of life. Despite promising findings, there is still insufficient high-quality clinical evidence to broadly recommend TM supplementation in oncology, and its use should be approached with caution until further research clarifies efficacy and safety.

Conversely, exposure to toxic elements has been strongly linked to cancer development through multiple, interrelated mechanisms [94,95,96]. One central pathway involves oxidative stress and chronic inflammation, both of which promote DNA damage, genetic instability, and aberrant cell signaling. Arsenic, Cd, and Pb interfere with DNA repair mechanisms, increasing the accumulation of mutations and facilitating malignant transformation. Arsenic and Cd also act as endocrine disruptors, altering hormone-regulated pathways involved in tumor progression. Additionally, Cd and Hg can promote epigenetic modifications, including DNA methylation and histone modifications, leading to the dysregulation of tumor suppressor genes and oncogenes. Cadmium and Pb contribute to abnormal cell proliferation and the inhibition of apoptosis, further enhancing carcinogenesis.

### 5.4. Neurodegenerative Diseases

Essential TMs are critical for the normal functioning and protection of the nervous system, playing crucial roles in neurotransmitter synthesis, antioxidant defense, and enzymatic regulation. Their presence in appropriate concentrations is fundamental for neuronal health, but both deficiencies and excessive accumulation can lead to severe dysfunctions. Neuroinflammation is an emerging concept in this context as a process that links TM imbalances to the progression of neurodegenerative disorders such as Alzheimer’s disease, Parkinson’s disease, and multiple sclerosis [97]. When TM levels deviate from optimal ranges, the nervous system becomes vulnerable to oxidative stress, inflammatory responses, and neuronal degeneration, contributing to the onset and worsening of these disorders.

Among the TMs that are most important for neurological function, Zn, Cu, Fe, Mn, and Se stand out for their essential roles in maintaining brain health. Zinc is fundamental for neurotransmission and synaptic plasticity, regulating the brain’s response to oxidative stress and inflammation [98]. While adequate Zn levels contribute to cognitive function, deficiencies have been linked to increased susceptibility to Alzheimer’s disease. Conversely, excessive Zn accumulation can disrupt neuronal homeostasis, leading to the aggregation of amyloid beta and exacerbating neurodegenerative processes [99]. Elevated Cu levels contribute to oxidative stress and neuroinflammation, accelerating neurodegeneration, as observed in Alzheimer’s and Parkinson’s diseases. By contrast, insufficient Cu levels can impair neuronal signaling and myelin formation, further compromising cognitive and motor functions [100]. Iron plays a critical role in oxygen transport and electron transfer, supporting neuronal metabolism. However, excessive Fe accumulation is a well-established factor in neurodegenerative diseases, particularly Alzheimer’s disease, in which it promotes the formation of reactive oxygen species, triggering oxidative stress and neuronal damage. In Parkinson’s disease, excess iron accumulates particularly in the substantia nigra, where it contributes to dopaminergic neuron loss through the Fenton-driven production of hydroxyl radicals and subsequent oxidative damage. The accumulation of Fe further exacerbates neuroinflammation, contributing to disease pathology [101]. Manganese, which is essential for enzyme activation and neurotransmitter production, must also be tightly regulated [102]. While necessary for brain function, chronic exposure to or excessive accumulation of Mn can result in neurotoxicity, leading to manganism, a condition resembling Parkinson’s disease. Studies have shown that high Mn levels induce oxidative stress and neuroinflammation, further highlighting the importance of maintaining Mn homeostasis. Selenium, known for its antioxidant properties, plays a protective role in preventing oxidative damage in the brain. It supports key enzyme systems, such as GPx, which neutralize reactive oxygen species and help maintain neuronal integrity. Selenium deficiency has been associated with cognitive decline and an increased risk of neurodegenerative diseases, reinforcing the importance of this TM in brain health [103].

Given the dual role of TMs as essential neuroprotective factors and potential neurotoxic agents, therapeutic strategies aimed at modulating TM levels in the brain are emerging as potential interventions for neurodegenerative diseases. Maintaining optimal TM homeostasis through diet, supplementation, or medical strategies could mitigate the effects of neurodegeneration, reduce inflammation, and improve clinical outcomes. Continued research is essential for developing precise therapeutic approaches that leverage the protective properties of TMs while minimizing their harmful effects, ultimately contributing to the prevention and management of neurodegenerative disorders.

Exposure to toxic elements has been implicated in the development and progression of neurodegenerative diseases through multiple converging mechanisms. Oxidative stress and neuroinflammation constitute a primary pathway, contributing to neuronal damage, protein misfolding, and synaptic dysfunction [104]. Lead, Hg, and Cd promote neuronal apoptosis and disrupt Ca homeostasis, impairing neurotransmission and neuronal plasticity [105]. Arsenic and Hg interfere with mitochondrial function, leading to energy deficits and increased neurotoxicity [106]. Additionally, Pb and Cd can alter epigenetic regulation, affecting the expression of genes involved in neuronal survival and synaptic maintenance [107]. Mercury has a strong chemical affinity for selenium, forming inert Hg–Se complexes that reduce the bioavailability of Se for selenoprotein synthesis. This sequestration impairs antioxidant defense systems and exacerbates neurodegeneration [108]. The combined effects contribute to cognitive decline and have been associated with neurodegenerative disorders such as Alzheimer’s disease, Parkinson’s disease, and amyotrophic lateral sclerosis, reinforcing the need to limit environmental exposure to these toxic metals.

The complex interplay between trace metals and neurodegenerative processes has been increasingly recognized as a critical factor in the onset and progression of disorders such as Alzheimer’s and Parkinson’s diseases. Trace metals, both essential and toxic, influence multiple pathological mechanisms, including neuroinflammation, oxidative stress, neurotransmitter dysfunction, protein aggregation, and epigenetic alterations. Table 3 summarizes the key neurodegenerative mechanisms alongside the trace metals implicated in each, based on current evidence. This overview highlights the multifaceted roles of these elements in neural health and disease, underscoring the importance of precise trace element assessment in understanding and potentially mitigating neurodegeneration.

### 5.5. Infectious Disease Response

Essential TMs such as Zn, Se, Cu, and Fe play pivotal roles in modulating immune system functions, thereby serving as essential components in the body’s defense against infectious diseases. These micronutrients are integral to the activation and regulation of both innate and adaptive immune responses, providing critical support in the development, differentiation, and function of immune cells. Balanced levels of these TMs ensure optimal immune surveillance and defensive capacities, facilitating the body’s ability to prevent, combat, and recover from infectious pathogens effectively [109]. Extensive research during the COVID-19 pandemic also highlighted the importance of essential TMs. The roles of Zn and Se are of particular note [110,111], as they are essential for enhancing antiviral immunity and mitigating the inflammatory responses that can be exacerbated during severe COVID-19 infections. Zinc and Se supplementation may offer benefits in both the prevention and adjunctive treatment of infectious diseases, particularly in populations at risk of deficiency, such as the elderly or patients with comorbidities. However, while promising, further well-controlled clinical trials are necessary to establish standardized supplementation protocols and confirm their efficacy in COVID-19 treatment.

Copper and Fe also play dual roles in supporting immune cell function and influencing inflammation, with Cu inhibiting viral replication and Fe being involved in oxygen transport and reactive oxygen species production [112]. The therapeutic use of these elements has been advocated for to improve recovery rates and reduce COVID-19 complications, suggesting that balanced TM levels are crucial for effective immune responses and clinical outcomes [112,113,114]. The strategic management of these micronutrients is therefore essential for optimizing immune function, ensuring effective responses to vaccination and treatment, and minimizing the impact of infection.

Exposure to toxic elements undermines immune responses and heightens susceptibility to infections by inducing oxidative stress and chronic inflammation [115]. These metals disrupt immune function; Cd and Pb alter cytokine production and inhibit lymphocyte proliferation, Hg promotes autoimmunity while suppressing defense, and As impairs macrophage activity, increasing the risk of infection. These immunotoxic effects, along with nutritional deficiencies, intensify disease severity in susceptible groups, and minimizing exposure to these metals is essential to preserve immune health and enhance recovery from infection [116].

## 6. Global Challenges of Trace Minerals: Deficiencies and Toxicities

The global landscape of TM nutrition is marked by a complex interplay of essential TM deficiencies, inadequate levels of micronutrients (including vitamins), and exposure to toxic elements. This situation leads to significant public health challenges across different socioeconomic and geographical contexts. A recent study modelling the dietary intake of 15 micronutrients (excluding fortification and supplementation), with data from 185 countries from the Global Dietary Database [117], estimated that over 5 billion people (approximately 68% of the global population) do not consume adequate amounts of I, vitamin E, or Ca. Similarly, deficiencies in Fe, Zn, and folate affect more than half of the global population, with significant variations related to age and gender. For instance, more than 50% of children under five years are deficient in either Fe, Zn, or vitamin A, with critical impacts on their cognitive and physical development. Among women of reproductive age, 66% are deficient in one or more key nutrients such as Fe, folate, and I, which can contribute to pregnancy complications and higher infant mortality rates.

Moreover, geographical factors significantly influence the prevalence of essential TM deficiencies and toxic exposures. In areas with soil naturally low in Se or I, widespread deficiencies can occur that affect population health across economic boundaries [118]. Conversely, in regions with heavy industrialization or inadequate environmental regulations, the risk of exposure to toxic elements may be heightened, complicating the public health landscape.

The challenge is further complicated by subclinical deficiencies, which are often not apparent through routine health assessments but can still have a profound impact on long-term health outcomes. Estimates suggest that subclinical deficiencies are twice as prevalent as the corresponding clinical conditions, indicating that a vast number of individuals are at risk of adverse health effects without overt symptoms [119].

This nuanced global situation calls for a comprehensive approach that considers the dual challenges of ensuring the adequate intake of essential minerals while also preventing and mitigating exposure to toxic elements. Such strategies must be adapted to the specific dietary, environmental, and economic conditions of each region, emphasizing the importance of targeted public health interventions and policies (Table 4).

## 7. Trace Minerals in Low-Income Countries: Scarce Essentials and High Toxics

In regions with limited resources, malnutrition and restricted access to mineral-rich foods are the primary contributors to essential TM deficiencies. Globally, many people living in these areas suffer from chronic micronutrient deficiencies, a condition collectively termed “hidden hunger” [120]. This concept highlights the subtle, often less visible signs of undernutrition that nonetheless have profound and long-lasting effects on health, cognitive development, and economic productivity.

The most prevalent TM deficiencies worldwide are Fe, Zn, and I deficiencies, together with vitamin A and folate deficiencies [121,122]. Together, these nutrient deficiencies contribute to approximately 7% of the global disease burden annually. According to the FAO, 372 million preschool-aged children and 1.2 billion women of childbearing age are deficient in at least one key micronutrient. A high proportion of these individuals (approximately 75%) reside in South and East Asia, the Pacific, and sub-Saharan Africa, where populations often experience multiple micronutrient deficiencies simultaneously.

Young children and women of reproductive age remain particularly vulnerable in low-income countries. The 2021 *Lancet* series on Maternal and Child Undernutrition [123] emphasizes that, despite modest advancements, undernutrition continues to be a significant global problem, further exacerbated by the COVID-19 pandemic. Vitamin A and Zn deficiencies still contribute to child mortality and the global burden of diseases, highlighting the critical need for effective nutritional interventions within the first 1000 days of life to combat these deficiencies and improve long-term health outcomes. Zinc deficiency increases infectious morbidity and reduces linear growth, while I and Fe deficiencies are primarily significant for their effects on development and cognition, leading to consequent disabilities.

The dietary patterns of low-income regions, predominantly based on cereals and tubers, with minimal animal-sourced foods, often fail to provide adequate levels of micronutrients [122]. In addition, agricultural practices that deplete soil mineral content exacerbate deficiencies, particularly in nutrients like Se and Zn, which rely on soil availability. These challenges are particularly pronounced in communities that depend heavily on locally produced food systems [124,125]. The issue extends beyond dietary diversity and adequacy, as affordability continues to pose a significant barrier to accessing a healthy diet. The FAO’s 2024 report reveals that over 70% of people in low-income countries cannot afford a nutritionally balanced diet, severely limiting their intake of essential minerals [122]. Furthermore, rising food prices and the impacts of climate change have driven shifts in dietary patterns, often favoring cheaper, nutrient-poor staples over foods rich in micronutrients. This trend increases the risk of hidden hunger, posing significant threats to global health and economic development [126]. Despite advancements in nutrition programs, several obstacles persist. Cultural resistance, logistical barriers, and low adherence rates hinder the effectiveness of interventions such as food fortification and supplementation programs [127]. These issues are particularly acute in rural and underserved areas, where implementing health and nutrition strategies is more challenging. Overcoming these barriers requires a comprehensive, multisectoral approach that integrates nutrition education, enhanced agricultural practices, and targeted supplementation to ensure reliable access to essential micronutrients [128].

Beyond the challenges of addressing essential micronutrient deficiencies, low-income regions also face significant health risks due to exposure to toxic elements originating from unregulated industrial activities, artisanal mining, and inadequate waste management, contaminating water supplies and soils [129]. Such exposure not only competes with the absorption and utilization of vital nutrients like Fe and Zn, exacerbating their deficiencies, but also directly impairs physiological functions, contributing to a range of health disorders. Exposure of children to Pb and Mn is of particular concern, as it is linked to neurocognitive impairments, indicating the urgent need for policies to mitigate these exposures and protect child development [130]. Cadmium can damage kidney function and bone structure, further complicating the health landscape in these vulnerable populations [130]. Arsenic exposure is linked to an increased risk of skin, lung, and bladder cancers, and its presence in drinking water, common in regions such as South Asia, illustrates the urgent need for interventions that address both nutritional deficiencies and toxic exposures [131]. The complex interplay between TM deficiencies and toxic element exposure requires integrated public health approaches that include environmental monitoring, community education on risk mitigation, and policies focused on reducing industrial emissions and improving waste management to safeguard soil and water quality [132]. Innovative research into low-cost technologies for heavy metal removal and enhanced regulatory frameworks are crucial to mitigate these risks, ensuring safer drinking water and healthier communities [133,134].

## 8. Trace Minerals in High-Income Countries: Nutritional Gaps and Toxic Risks in an Aging Society

Essential TM deficiencies persist in high-income regions, largely driven by poor dietary choices influenced by socioeconomic disparities and lifestyle factors, rather than by absolute scarcity. In a predominantly aging population, these nutritional deficits can have important health implications [135]. According to the World Health Organization [22], Fe deficiency is the most prevalent TM imbalance in these countries; it is very common among women of reproductive age and young children, contributing to widespread anemia and related health complications, despite overall food security. These problems are compounded by diets that heavily feature processed foods that tend to be low in essential micronutrients such as Fe, Ca, and Zn, necessary for metabolic health and disease prevention [136]. Extensive studies of dietary dynamics in high-income countries have shown that although ultra-processed foods can provide most of the caloric requirements, they are generally low in Fe and Zn, which are crucial for metabolic health and disease prevention [137]. These researchers emphasize the importance of enhancing diet quality by increasing the intake of nutrient-dense foods, including animal-source foods (such as organ meats, small fish, and dairy products), which are rich in bioavailable micronutrients, and certain plant foods such as dark green leafy vegetables, which are also important for meeting micronutrient requirements [138,139].

The impact of diet quality on TM intake in high-income countries is further exemplified by the growing uptake of vegetarian and vegan diets. While these diets are celebrated for their health benefits, they can also lead to a risk of deficiencies in essential minerals such as Fe, Zn, I, Se, and Ca. These minerals, which are essential for immune function, bone health, thyroid regulation, and overall metabolic processes, are typically more concentrated and bioavailable in animal-based foods [140]. For example, plant-based Fe, or non-hem Fe, is absorbed less efficiently than the hem Fe found in animal products [141]. Consequently, studies have reported lower serum ferritin levels in vegans, indicating an increased risk of Fe-deficiency anemia [142,143]. Calcium intake can also be deficient, as vegan diets exclude dairy products, one of the most bioavailable sources of Ca. This can result in lower bone mineral density and a heightened risk of fractures among vegans than in omnivores [144]. Similarly, Zn absorption can be compromised in vegan diets due to the presence of phytates in plant-based sources like whole grains and legumes, which inhibit Zn uptake [145]. Iodine deficiency is also of concern, particularly where iodized salt is not routinely used, potentially leading to thyroid issues in vegan populations [146]. Furthermore, crops grown in Se-deficient soils pose additional risks for vegans, as Se deficiency affects antioxidant defense and thyroid function, highlighting the critical link between dietary choices, the local environment, and nutrient availability [41,147].

The adequate intake of TMs can be challenging in vegans and individuals who adopt dietary patterns such as the EAT-Lancet diet [148], which also advocates for a large reduction in animal product consumption and faces similar issues. Proposed in 2019 by the EAT-Lancet Commission, this diet promotes a plant-based eating pattern with a substantial reduction in animal products to improve human health and reduce environmental impact. Although not as restrictive as the vegan diet, it shares some of the risks of TM deficiencies due to a limited inclusion of animal sources rich in essential nutrients like Fe and Zn [149]. These TMs are crucial for metabolic health and are not as bioavailable in plant sources. Studies have shown that reducing animal products in the diet may lead to a lower intake of essential minerals, especially in populations already facing low nutrient availability. Moreover, a global analysis of the affordability of the EAT-Lancet diet suggests that many low-income populations may face difficulties accessing plant-based and fortified foods that compensate for animal sources of minerals [149]. The reduction in meat and dairy consumption limits the intake of hem Fe and Ca, which are more bioavailable from animal sources. Iron deficiency, in particular, could become a problem if the diet is not adequately supplemented with vitamin C-rich foods to enhance the absorption of non-hem Fe from plants. These findings illustrate the importance of monitoring mineral status in individuals following the EAT-Lancet diet and considering micronutrient supplementation to prevent deficiencies similar to those that may arise in strictly vegan diets [137,149].

In high-income countries, dietary challenges are compounded by social factors such as an aging population, which has an increased demand for micronutrients but tends to exhibit poor dietary habits due to a selective appetite and reduced food intake [150]. This demographic is particularly vulnerable to micronutrient deficiencies because of natural age-related decreases in nutrient absorption [151] and also the prevalence of chronic diseases. Chronic conditions are often linked to, and can be exacerbated by, an inadequate micronutrient status, thus highlighting the critical need for targeted nutritional strategies. Ensuring adequate TM intake in elderly populations is essential, given that deficiencies can significantly impact their overall health and exacerbate the progression of chronic illnesses [152]. This calls for a comprehensive approach that addresses both dietary improvements and the broader social factors influencing the nutritional status in these communities [153].

In high-income countries, exposure to toxic elements leads to serious challenges associated with a variety of industrial, consumer, and environmental sources [154]. Despite strict regulations and advanced detection and remediation technologies, the presence of these metals in the environment continues to impact public health. Major sources of exposure include drinking water and food, especially in the case of As, which can be found in high concentrations in groundwater and in foods such as rice [60]. Tobacco consumption is a primary source of Cd [155], along with certain industries that release Cd as a by-product. Although the use of Pb has been restricted, its legacy persists in old infrastructures, lead-based paints, and water pipes, with children mainly being affected, including by developmental problems [63]. Fish and seafood are the primary sources of Hg, especially in deep-water species like tuna and swordfish [66]. Dietary trends, such as an increased consumption of fish in certain diets, can heighten exposure to Hg, while the predominantly aging population may be particularly vulnerable to the toxic effects of these metals due to decreased detoxification capacities.

Exposure to toxic elements is exacerbated by globalization, which leads to imports of potentially contaminated products from regions with less strict regulations [156]. In addition, historically contaminated sites continue to be a source of concern, requiring remediation efforts and focused public policies [157]. Socioeconomic disparities also play a role in differential exposure to toxic elements. Despite generally high food security and strong regulations, some communities within high-income countries, particularly economically disadvantaged people, may face greater risks of exposure due to their proximity to industrial areas or restricted access to safe food and clean water [158]. These challenges highlight the importance of ongoing surveillance, strengthening environmental regulations, and implementing more effective remediation technologies. It is also important to promote a greater awareness of the risks of exposure to toxic metals and to improve public education to mitigate these risks. This complex landscape in high-income countries requires a comprehensive approach that not only addresses direct sources of exposure but also considers the social and public health implications of toxic metal contamination.

## 9. Trace Minerals in Middle-Income Countries: Dual Challenges of Nutrition and Toxicity

In middle-income countries, essential TM deficiencies often reflect a complex interplay of socioeconomic factors that create a dual burden of malnutrition [159]. These nations exhibit considerable economic and social heterogeneity, which in turn affects dietary patterns across different social strata and geographical areas. In urban areas and among higher economic classes, there is a notable prevalence of ultra-processed food consumption, mirroring patterns observed in high-income countries [160]. Such diets are typically high in energy but low in essential micronutrients, contributing to what is referred to as ‘hidden hunger’—whereby individuals consume sufficient calories but insufficient nutrients. This phenomenon often coexists with lifestyle factors such as sedentary habits and high rates of smoking and alcohol consumption, exacerbating the risk of micronutrient deficiencies and associated health disorders. Conversely, in rural and less economically developed regions, the primary concern is often the lack of access to nutritious foods—a challenge more commonly associated with low-income countries. Here, populations may struggle with insufficient dietary diversity and limited availability of food rich in essential vitamins and TMs, leading to overt malnutrition and nutrient deficiencies [161].

Exposure to toxic elements in such regions also reflects the complex interactions in socioeconomic factors contributing to distinctive environmental risks [162]. These countries face unique challenges due to their economic and social heterogeneity, which affects industrial practices and regulatory policies. In many urban areas and among higher economic classes, rapid industrialization and urban growth have led to significant problems related to air and water pollution, with major sources of toxic metals such as Pb and Hg originating from factories and motor vehicles [163]. These issues are exacerbated by infrastructure that is often insufficient to effectively manage industrial and urban waste, resulting in the continuous release of toxic elements into the environment. On the other hand, in rural and less economically developed parts of these countries, exposure to pesticides and chemical fertilizers used in agriculture can be a prominent source of toxic elements such as Cd and As [164]. The lack of strict regulations and effective enforcement allows these practices to continue, impacting both human health and food safety.

In middle-income countries, addressing both essential nutrient deficiencies and toxic exposure requires a careful, multifaceted approach due to diverse socioeconomic disparities [165]. Strategies must include enhancing access to and education about nutrient-dense foods, particularly in areas where unhealthy diets prevail, and utilizing food fortification and regulatory measures to promote healthier eating habits. Enhancing regulations in industrial, agricultural, and waste management, along with boosting environmental monitoring and compliance, is essential for reducing exposure to toxic elements. Supporting local agriculture and the distribution of locally produced, nutrient-rich foods both improves nutrition and bolsters local economies. By tailoring strategies to specific regions and demographics, middle-income countries can more effectively navigate the intricate challenges of nutritional and environmental health, thereby enhancing public health and environmental sustainability. The WHO highlights the importance of such integrated approaches in addressing global health and environmental challenges [166].

## 10. Trace Mineral Assessment of the Population

TM levels in populations are often estimated through an assessment of dietary intake. Globally, several organizations have established frameworks to guide both essential TM intake and monitor exposure to toxic elements. In Europe, the European Food Safety Authority (EFSA) plays a leading role in assessing dietary needs and risks associated with nutrient consumption, as well as evaluating the risks posed by toxic elements. In the United States, the National Research Council (the operating arm of the National Academies of Sciences, Engineering, and Medicine, NASEM), formerly known as the Institute of Medicine (IOM), and in Canada, the National Research Council Canada, also provide comprehensive dietary recommendations for essential TM and risk assessment frameworks for toxic elements. Internationally, the Food and Agriculture Organization (FAO) and the World Health Organization (WHO) have developed dietary guidelines, particularly aimed at addressing deficiencies and managing toxic exposure in developing countries. However, FAO/WHO recommendations tend to be less frequently updated and may not account for recent advancements in nutritional science, which limits their applicability to current challenges in both nutrition and contamination control [167,168].

All of these agencies use specific parameters to define the recommended intakes of essential TMs for different populations, tailored by age, sex, and physiological status, including pregnancy and lactation (for a detailed review, see [167,168]). Commonly used values include the *average requirement*, which is the intake level estimated to meet the needs of 50% of a specific population group, the *recommended intake*, derived from the average requirement, which is the level sufficient to meet the needs of nearly all (97–98%) individuals in a group, and the *tolerable upper level*, which is the maximum intake unlikely to pose risks of adverse health effects in the general population [169]. When precise data are not available for establishing an average requirement, *adequate intake* values, based on observed or experimentally determined estimates of nutrient intake by a group of healthy people, can be used.

For toxic elements, the above organizations establish health-based guidance values such as *tolerable weekly intakes* and *tolerable daily intakes* to manage risks. Tolerable weekly intakes are typically used for substances that accumulate in the body, ensuring that long-term exposure remains within safe limits, while tolerable daily intakes are often applied to control short-term exposures. The adoption of the *Benchmark Dose* methodology marks a significant advancement in risk assessment [170]. This method, preferred over the traditional No-Observed-Adverse-Effect Level (NOAEL), involves modeling dose–response data to determine a dose that produces a predefined change in the response rate, typically a small but measurable adverse effect. This shift reflects a more refined scientific approach to establishing exposure thresholds, enhancing the accuracy and reliability of health risk assessments. Additionally, the margin of exposure (MOE) approach is used to assess the risks associated with carcinogens for which no safe exposure levels have been determined, providing a quantitative measure of how much the exposure level in humans exceeds an established risk level from animal or human data [155].

Following the establishment of guidelines for essential and toxic TMs by these agencies, assessing whether populations meet requirements or are exposed to high levels of TMs generally involves an indirect method through dietary intake assessment. Such assessment is commonly based on methods such as 24 h recalls, food diaries, and frequency questionnaires, where the consumption data collected are combined with tabulated data on the concentrations of both essential and toxic TMs in each consumed food. This approach is beneficial due to its lower cost and ease of implementation on a large scale, providing valuable data on population-wide eating habits. However, it has several limitations [171,172]. The tabulated data on nutrient and contaminant content are often not updated frequently and may overlook regional variations in food composition and bioavailability, as well as factors such as soil mineral content and food fortification practices. These limitations can significantly affect the accuracy of the assessment. Additionally, dietary intake assessment relies heavily on participant honesty and memory, which can introduce reporting bias. Thus, the calculations derived from this approach may not always be precise, impacting the effectiveness of nutritional assessments and the development of dietary recommendations.

It is important to acknowledge that this method does not account for numerous factors that influence an individual’s essential TM status and accumulation of toxic elements. Factors related to the bioavailability of TMs must be considered. These factors vary greatly depending on whether the minerals come from animal or plant sources [143] and the health status of individuals, which plays a critical role, particularly in aged populations in developed countries where chronic diseases are prevalent [4]. Conditions such as chronic inflammation, obesity, and sedentary lifestyles can markedly affect the absorption and metabolism of TMs. These health-related factors can lead to altered mineral status independently of dietary intake. In elderly populations, the presence of chronic diseases often necessitates a more detailed approach to nutritional assessment, as these conditions can impair nutrient assimilation and utilization [173].

The EFSA, which has established dietary reference values for essential TMs, consistently highlights critical knowledge gaps that limit the accurate assessment of TM status in the population. A major concern is the lack of sensitive and specific biomarkers for essential TM such as Cu, Se, Fe, and Zn [8,9,11,12]. Without reliable indicators, it is difficult to determine the true prevalence of deficiencies or to assess their health consequences. For toxic elements, the EFSA also emphasizes the need for improved methods to measure the internal exposure and evaluate the associated risks [60]. There are unique challenges associated with these elements due to the potential for bioaccumulation, non-dietary sources, and the long delays in health effects, which often require longitudinal data for detection. To address these limitations, further research is needed to better understand the metabolism, bioavailability, and physiological regulation of both essential and toxic TMs across various life stages and health conditions, including pregnancy, lactation, aging, obesity, and chronic low-grade inflammation. For Cu and Se, studies should explore the influence of genetic polymorphisms—such as those affecting selenoprotein expression—on bioavailability and health outcomes. Iron-related research should refine current knowledge on homeostasis and absorption within the context of whole diets, while Zn studies must consider inhibitory factors like phytates, particularly in plant-based diets. The interactions between toxic elements and essential nutrients must also be further investigated in greater detail, as these can alter absorption and metabolism, complicating risk assessment. Ultimately, overcoming these scientific and methodological gaps requires the development of standardized, validated biomarkers capable of accurately reflecting both essential TM status and toxic exposure. Such biomarkers are essential for improving the precision of nutritional surveillance, guiding risk management strategies, and supporting effective, evidence-based public health interventions.

## 11. The Need for Biomarkers in Assessing Mineral Status

While indirect methods of dietary intake assessment are indispensable for broad epidemiological studies and monitoring trends at the population level, providing insights into dietary patterns and potential risk areas, they lack the precision needed to fully account for bioavailability and individual variability [174]. By contrast, direct methods using biomarkers of exposure provide unmatched detail. Such biomarkers are particularly relevant in contemporary societies where dietary patterns are influenced by factors such as veganism, obesity, aging, and the prevalence of chronic diseases. These factors introduce significant variability in TM intake, absorption, and metabolism, making precise measurements crucial for effectively tailoring nutritional interventions and reducing exposure to toxic elements [171]. As already mentioned, although vegan diets may provide adequate calories, they often lack bioavailable forms of essential TMs like Fe and Zn, necessitating targeted supplementation. Similarly, aging populations may experience decreased absorption efficiency and altered nutrient requirements, which cannot be adequately captured through indirect dietary assessments alone. Furthermore, biomarkers are invaluable for monitoring exposure to toxic elements. These toxic elements can accumulate in the body over time from various sources, not only through diet but also from lifestyle choices and environmental factors. For example, Cd exposure is greatly influenced by smoking, as tobacco smoke is a major source of this toxic metal [175]. Similarly, exposure to Hg can be significantly elevated by the use of fossil fuels, particularly in areas near coal-fired power plants, which are a major source of Hg emissions [176]. These biomarkers provide critical insights into the total burden of toxic elements, facilitating more accurate risk assessments and enabling interventions to mitigate their health impacts. 

The use of biomarkers is also important for identifying the subclinical deficiencies of essential TMs or exposure to toxic elements, whereby individuals may not exhibit obvious symptoms but could still be impacted over time [3]. The subclinical deficiencies of essential TMs are increasingly recognized as a public health concern, as they can contribute to fatigue, immune dysfunction, and cognitive impairment without immediate clinical signs [177]. By leveraging both approaches—indirect dietary assessment and direct biomarker analysis—public health initiatives can more effectively address nutritional deficiencies and manage the risks associated with toxic element exposure, promoting overall health across diverse populations.

The ideal biomarker for assessing TMs should have several definitive characteristics to ensure accurate and practical use in nutritional and epidemiological evaluations [178]. Specificity is paramount; the biomarker must distinctly reflect the levels of a specific TM and be unaffected by other substances or underlying conditions. Sensitivity is critical for detecting both excess and deficiency early, often before clinical symptoms become apparent. Consistency is also essential; results should be reproducible across various testing scenarios and different laboratories, ensuring reliability. The biomarker must be responsive, capable of quickly reflecting changes in micronutrient levels due to dietary adjustments or health status changes. For practical implementation, especially in population-wide studies, the biomarker should ideally be obtained through non-invasive or minimally invasive means to facilitate easy, widespread collection. Cost-effectiveness enhances the applicability of biomarkers in diverse economic settings, making the methods accessible for routine use in both developed and developing regions. Finally, an ideal biomarker should provide actionable insights, aiding healthcare professionals and policymakers to make informed decisions for addressing micronutrient-related health issues.

The biomarkers of TMs can generally be categorized into two main types: those that measure the concentrations of TMs in a biological matrix, such as blood, urine, and hair, and those that evaluate parameters related to the element’s function or impact within the body. For essential TMs, this includes their involvement in enzymes, hormones, functional indices, or storage sites, reflecting their roles in metabolism and homeostasis. Conversely, for toxic elements, biomarkers measure both recent exposure or chronic accumulation and their physiological effects, such as organ function impairment and systemic damage, reflecting the potential health impacts of their presence and accumulation in the body.

The primary biomarkers available for essential TMs are summarized in Table 5. Unfortunately, most of these endpoints do not meet all of the criteria of an ideal biomarker because the effectiveness of a biomarker largely depends on the physiological and toxicokinetic properties of the element it measures. For essential elements, homeostatic regulation and the characteristics of the biological matrix may limit their ability to reflect the true status across the continuum from deficiency to excess. For toxic elements, factors such as bioaccumulation, tissue distribution, and the timing of exposure can affect the capacity of blood biomarkers to accurately indicate total body burden or potential health risks.

The homeostasis of essential TMs such as Se and I predominantly relies on renal excretion, with limited regulatory mechanisms at the level of intestinal absorption [179] controlled by urinary elimination. This physiological characteristic enhances the diagnostic value of certain biomarkers used to assess their status. In the case of Se, functional markers such as GPx and selenoprotein P (SePP) are highly sensitive to deficiency states, as they directly reflect the body’s antioxidant capacity [180]. However, the utility of these markers decreases once Se levels are sufficient and the responses plateau, making them less effective for detecting excessive intake. By contrast, serum Se concentrations continue to increase proportionally with dietary intake, making serum measurements more reliable for assessing overexposure, which is particularly relevant in regions with Se-rich soils [181]. For I, urinary concentration has long been considered the gold standard biomarker due to its strong correlation with thyroid function, where I plays an essential role [182]. This is largely attributable to the ease of its analytical determination—typically performed using colorimetric techniques such as the Sandell–Kolthoff reaction—and the non-invasive nature of urine sampling. Nonetheless, accurate assessment requires either 24 h urine collection or correction for urinary creatinine in spot samples to account for dilution-related variability. Recent studies suggest that serum I concentrations may also provide valuable insights, especially when a fasting serum sample is already being collected for other analyses, and particularly in the context of a comprehensive mineral profile obtained through multi-element techniques such as ICP-MS [183].

By contrast, elements such as Fe, Zn, Cu, and Cr are primarily regulated at the level of intestinal absorption and endogenous secretion [4], rather than by renal excretion. As a result, urinary concentrations have limited value as biomarkers of status. In the case of Fe, serum levels are often considered unreliable due to their susceptibility to confounding factors such as hemolysis and systemic inflammation [184]. Functional indices, including hematocrit, hemoglobin concentration, and mean corpuscular volume (MCV), which are commonly used in the diagnosis of Fe-deficiency anemia, are more informative. However, these parameters lack specificity, as they can also be altered in non-nutritional forms of anemia. Ferritin is widely used to estimate Fe stores and support diagnosis. According to the WHO [185], ferritin is a good marker of Fe status in apparently healthy individuals, but its interpretation is limited in the presence of inflammation, liver disease, obesity, and malignancy. In such cases, ferritin levels may be elevated regardless of Fe status, and the concurrent measurement of acute-phase proteins (e.g., CRP and AGP) is recommended to adjust or interpret ferritin concentrations correctly. The measurement of the reticulocyte hemoglobin equivalent (RET-He), included as a marker in most new hematology analyzers, can potentially be used to detect Fe deficiency and Fe-deficiency anemia [186]. For Zn and Cu, serum concentrations are routinely measured and can reflect deficiency under conditions of low intake [9,12]. Nonetheless, once the physiological requirements are met, these concentrations plateau and no longer correlate well with dietary intake. Functional biomarkers such as fatty acid desaturase activity (for Zn) [187] and ceruloplasmin oxidase activity (for Cu) [188] have been proposed to improve the accuracy of assessment, although further validation is still required before routine clinical implementation.

For toxic elements (Table 6), the biomarkers of exposure often include measurements of the element in blood, urine, or other tissues, depending on their toxicokinetic profiles. Lead exposure, for instance, is commonly assessed via blood Pb levels, which are reliable indicators of both recent and cumulative exposure [189].

Mercury exposure can be monitored through an analysis of blood or hair, with hair concentrations reflecting long-term exposure, particularly to methylmercury from dietary sources such as fish [190]. Cadmium exposure is typically evaluated by measuring Cd levels in urine, reflecting both recent and cumulative exposure due to its long biological half-life; however, Cd can also be detected in blood, especially to assess recent intake [191]. Similarly, As exposure is often assessed by measuring total As in urine, which captures all forms of As and offers a comprehensive overview of exposure, although blood As can also be useful in specific contexts [192]. In addition to exposure biomarkers, biomarkers of effect—such as δ-aminolevulinic acid dehydratase (ALAD) inhibition for Pb or increased micronucleus frequency for genotoxicity—provide valuable insights into the biological impact of these elements and help assess potential health risks [193].

There is no single ideal biomarker suitable for assessing the status of all trace and toxic elements. The selection of an appropriate biomarker largely depends on the specific element of interest and the clinical or public health question being addressed. When the objective is to evaluate the status of a particular element—whether to detect deficiency or excess—biomarkers should be selected on the basis of their sensitivity and specificity for the context. For example, serum ferritin is commonly used to assess Fe stores, while blood Pb levels are preferred for evaluating Pb exposure. In cases of suspected deficiency, functional biomarkers may also be useful, such as GPx for Se deficiency. However, in population-based studies or environmental surveys where the aim is to assess a broad range of both essential and toxic elements, the matrix chosen should provide the best compromise between feasibility and diagnostic value. In this context, serum represents a particularly valuable option: it is widely accessible, well-characterized for most essential elements, and informative for many toxic metals when analytical sensitivity is adequate. Given that the most common trace element disorders in humans include Fe, Zn, Se, and I deficiencies, along with exposure to toxic metals such as As, Cd, Hg, and Pb, serum analysis can provide highly relevant epidemiological insights. Although whole blood remains the gold standard for some toxic elements due to the specific distribution of these in blood compartments, serum measurements can serve as a reliable starting point for public health screening and surveillance efforts. Its value lies not only in its compatibility with high-sensitivity multi-element techniques but also in its practicality: serum samples are routinely collected in clinical settings and can be processed using protocols already established for other diagnostic purposes. Additionally, serum allows the simultaneous monitoring of both essential and toxic elements within the same matrix, facilitating integrated nutritional and toxicological assessments. These characteristics support its potential as a cornerstone matrix for implementing broader micronutrient surveillance and personalized nutrition strategies.

## 12. The Power of Micromineral Serum Analysis in Public Health Interventions

Serum TM analysis is not a recent innovation. Its origins can be traced back to the mid-20th century, when increasing scientific interest in the biological role of micronutrients prompted the development of analytical techniques capable of detecting these elements at low concentrations. A breakthrough occurred in 1955, when Alan Walsh introduced the principle of atomic absorption spectroscopy (AAS), which enabled the quantitative determination of metal ions in biological samples for the first time [194]. Despite this milestone, early applications of AAS and conventional spectrophotometry were limited by insufficient sensitivity and multi-element capacity, restricting the practical use of these methods in large-scale studies. However, the field underwent a major transformation with the advent of inductively coupled plasma mass spectrometry (ICP-MS), a multi-element measurement technique that allows the simultaneous determination of almost all elements, both toxic and essential, of interest to humans [195]. The ability to detect element concentrations in the order of parts per trillion revolutionized the field. The exceptional sensitivity of this method, combined with rapid analysis capabilities and decreasing costs, has enabled simpler, more precise, and economically viable serum micromineral analysis. The use of ICP-MS has expanded greatly in epidemiological and clinical research, enabling a more detailed exploration of the relationship between TMs and health. Furthermore, improvements in technician training and technology accessibility have facilitated its broader adoption across a wider range of laboratories, promising future innovations that could further enhance these analyses. However, despite these scientific and technical advances, the implementation of serum TM analysis in routine, non-research clinical settings remains limited. Barriers include the high cost of instrumentation and maintenance, the need for trained personnel, and the absence of harmonized clinical guidelines and validated reference intervals for many elements. These factors continue to pose a significant obstacle to the widespread clinical integration of trace element monitoring.

In addition to analytical instrumentation, proper sample collection and handling are essential to ensure accurate and reproducible trace element measurements in serum. Blood must be drawn using certified trace element-free tubes to avoid contamination, and hemolysis should be prevented during processing. After centrifugation, serum should be aliquoted into metal-free containers and stored at −80 °C until analysis. These precautions are particularly important for elements present at ultra-trace levels, where even minor contamination or degradation can significantly alter results. Accurate quantification also requires rigorous quality control protocols throughout the analytical process. This includes the use of certified reference materials, internal standards, blank corrections, and participation in external quality assessment schemes to ensure the comparability and traceability of results across laboratories.

The future of ICP-MS looks promising, with ongoing advancements enhancing its sensitivity and expanding its capacities. Combining ICP-MS with emerging technologies such as Single-Particle ICP-MS (SP-ICP-MS), Laser-Induced Breakdown Spectroscopy (LIBS), and Laser Ablation ICP-MS is set to broaden the applications even further [196]. Additionally, coupling ICP-MS with bioinformatics and digital health systems could revolutionize the personalization of nutritional assessments and health interventions, utilizing huge datasets and artificial intelligence to refine and tailor health strategies [197]. These developments are not just enhancing the precision and efficiency of TM analysis but are also paving the way for its integration in more dynamic and responsive public health initiatives.

The significant analytical advancement represented by ICP-MS has revolutionized serum analysis for detecting TMs, providing essential tools not only for identifying nutritional deficiencies and toxicities at both individual and population levels but also for early intervention. The feasibility of implementing serum multi-elemental analysis using ICP-MS in clinical settings has been increasingly documented in recent years. For instance, Kojo et al. [198] conducted a large comparative study demonstrating that ICP-MS yields accurate and reproducible results for key serum minerals, showing strong agreement with standard hospital-based methods and confirming its utility for detecting both deficiencies and excesses under real-world conditions. Similarly, Laur et al. [6] further emphasized the analytical performance and clinical applicability of ICP-MS for simultaneous trace element quantification in serum and whole blood, supporting its integration into clinical workflows and population-level screening. By detecting mineral imbalances before the manifestation of chronic diseases such as diabetes mellitus, cardiovascular diseases, neurodegenerative disorders, and some cancers, serum analysis enables preventive interventions that can significantly reduce the prevalence and severity of these conditions. Altogether, this proactive approach underscores the crucial role of serum analysis in preventive healthcare.

The precision of serum TM analysis also extends to the realm of personalized medicine [199]. By providing a detailed profile of an individual’s micronutrient status, clinicians can design tailored interventions that address each patient’s unique nutritional needs. The personalized approach not only enhances the effectiveness of treatments but also minimizes the risk of adverse effects associated with over-supplementation. Careful management is particularly important for elements like Se, which plays essential roles in immune function, inflammation regulation, and antioxidant defense but has a narrow safety margin [200]. Accurate Se supplementation, backed by meticulous serum analysis, is essential to ensure that individuals receive the optimal amount without risking toxicity. This precision is vital for older adults and patients with chronic organ diseases, who may be particularly susceptible to the consequences of both deficient and excess concentrations of the element. Public health initiatives that incorporate such personalized strategies based on detailed micronutrient assessments have consistently demonstrated improved patient outcomes and enhanced cost efficiency [201].

At the population level, the multi-element quantification of essential trace and toxic elements through serum analysis serves to monitor the impact of dietary changes and environmental exposure. The uptake of diets such as vegan diets [202] and the inclusion of new foodstuffs [203] can significantly alter the micronutrient profile, elevating exposure to toxic metals like As and Hg, which are predominantly found in some seafood products [204,205]. While these dietary shifts are beneficial from nutritional and ethical perspectives, they necessitate vigilant monitoring to pre-empt nutritional deficits and prevent toxic accumulation [206]. Moreover, modern urbanization and technology expose populations to novel environmental hazards, including heavy metals from water pollution [207] and the use of electronic devices [208]. This type of exposure raises significant concerns, especially for vulnerable groups such as pregnant women and children, where metal toxicity can severely impair fetal development and child health [209,210]. In this context, the role of elements such as Se in modulating Hg toxicity is important [211]. The accurate serum analysis of Se, and of other toxic metals, provides a comprehensive understanding of the nutritional benefits and risks associated with specific lifestyle and dietary changes. Serum analysis is thus an important way of evaluating the effectiveness of public health interventions aimed at adjusting or improving diets and living environments. Such information is essential for designing policies and programs that promote a healthy balance of micronutrients while minimizing exposure to toxic substances, thereby ensuring broader population protection.

## 13. Overcoming Challenges in Serum Trace Element Analysis for Future Healthcare

The technical aspects of serum TM analysis have advanced considerably, enabling very low limits of quantification to be reached with excellent analytical precision. In addition, these techniques are now affordable, and their availability in many laboratories facilitates integration in healthcare systems alongside other routine medical tests.

However, one of the main current limitations is the lack of widely accepted reference values for essential TMs that adequately reflect the diversity of global populations. It is essential to be able to differentiate between deficient, adequate, and potentially harmful concentrations of these elements. The ranges of these values can vary considerably across different regions due to differences in dietary habits, environmental exposure, and genetic factors. To address this knowledge gap, numerous studies worldwide have sought to define reference intervals with appropriate lower and upper limits, as summarized in Table 7. There is also a closely linked need to understand the biological variability in TM concentrations. These levels are influenced by age, sex, and metabolic differences—which may result in distinct patterns between genders and across life stages—and also by socioeconomic conditions and lifestyle factors such as smoking and dietary patterns. Improving the understanding of these sources of variability is key to enabling the design of more effective, individualized health interventions.

To move beyond the limited “snapshot” approach used in many studies, integrating longitudinal data collection is essential. This would enable the precise definition of reference intervals and provide insights into how TM levels change over time in response to lifestyle, dietary, and environmental shifts. The high variability observed in global datasets highlights the need for a robust, standardized international database that can support both targeted public health policies and the development of personalized medical strategies. These efforts—grounded in precise assessments of essential TM status and toxic element exposure—are fundamental to enable the full potential of serum TM analysis to be realized in future healthcare.

## 14. Conclusions

Trace mineral imbalances—whether due to deficiency, excess, or toxic exposure—represent a critical yet often overlooked dimension of global public health. The complex interactions between TMs and chronic diseases, aging, immune function, and environmental factors highlight the urgent need for precise, individualized, and population-level assessment strategies. Although dietary intake assessments offer important information, they are limited in their ability to account for individual variability, nutrient bioavailability, and non-dietary sources of exposure. In this context, serum analysis emerges as a powerful, practical tool. It provides reliable biomarkers for most essential trace minerals and, when supported by sufficiently sensitive analytical methods, can provide meaningful information about toxic metal exposure. The advent of high-throughput, multi-element measuring techniques such as ICP-MS has transformed serum analysis into a feasible and scalable approach, suitable for both clinical practice and large-scale public health surveillance.

Given the prevalence of Fe, Zn, Se, and I deficiencies and the widespread exposure to toxic metals like Pb, Cd, Hg, and As, serum-based monitoring aligns well with the most pressing trace element challenges across human populations. Although whole blood may remain the preferred matrix for certain toxic elements, serum provides the best compromise when aiming to assess a broad range of essential and harmful elements simultaneously.

Moreover, serum analysis is adaptable across diverse socioeconomic contexts: in low-income countries, it can support efforts to detect and address hidden hunger and early exposure to toxic elements; in middle-income regions, it helps navigate the dual burden of undernutrition and environmental contamination; in high-income settings, it facilitates the early detection of subclinical imbalances and provides information enabling the design of personalized strategies to support healthy aging and chronic disease management.

To fully harness the potential of serum trace element analysis, future efforts must focus on establishing reference values, standardizing methodologies, and integrating this approach into routine health assessments and precision nutrition strategies. By doing so, we can enhance risk stratification, guide targeted interventions, and thus contribute meaningfully to preventive and personalized public health frameworks.

## Figures and Tables

**Table 1:** Essential trace mineral functions and mechanisms in the body.

	Main Functions	Key Enzymes/Proteins/Mechanisms	Refs.
Fe	Oxygen transport (hemoglobin); immune function; energy production.	Hemoglobin, myoglobin, cytochrome c (electron transport chain), ferritin (storage), transferrin (transport), hem enzymes (e.g., catalase)	[8]
Zn	Protein and DNA synthesis; immune function; wound healing.	Zinc finger proteins (gene expression), carbonic anhydrase, alcohol dehydrogenase, superoxide dismutase (Cu/Zn-SOD) (antioxidant), metallothionein (regulation and storage)	[9]
I	Thyroid hormone synthesis; regulation of metabolism and growth.	Thyroid peroxidase (TPO), iodothyronine deiodinases, thyroglobulin (precursor protein)	[10]
Se	Antioxidant defense; thyroid function; immune support.	Glutathione peroxidases (GPx), thioredoxin reductase, selenoprotein P (selenium transport), iodothyronine deiodinases (thyroid hormone activation), selenoprotein W (muscle protection)	[11]
Cu	Energy production; connective tissue formation; brain function; iron metabolism.	Cytochrome C oxidase, superoxide dismutase (Cu/Zn-SOD), ceruloplasmin (iron oxidation), lysyl oxidase (collagen cross-linking), tyrosinase (melanin synthesis)	[12]
Cr	Blood glucose regulation; carbohydrate and lipid metabolism.	Chromodulin (enhances insulin receptor activity), a low-molecular-weight chromium-binding substance	[13]
Mn	Carbohydrate, protein, and lipid metabolism; antioxidant defense.	Manganese superoxide dismutase (MnSOD), arginase (urea cycle), pyruvate carboxylase (gluconeogenesis), glutamine synthetase (amino acid metabolism)	[14]
Mo	Amino acid metabolism; enzymatic function.	Xanthine oxidase (purine metabolism), aldehyde oxidase, sulfite oxidase (sulfur metabolism), molybdopterin-dependent enzymes	[15]

**Table 2:** Mechanisms of toxicity of selected toxic trace elements.

	Main Mechanisms of Toxicity	Target Systems/Effects
Cadmium	Oxidative stress, inhibition of Zn enzymes, mitochondrial dysfunction	Kidney dysfunction, bone demineralization, carcinogenesis
Lead	Disruption of Ca signaling, oxidative stress, neurotoxicity	Neurodevelopmental impairment, anemia, bone toxicity
Mercury	Sequestration of Se, inhibition of antioxidant enzymes, neurotoxicity	Neurological damage, cognitive decline, cardiovascular risk
Arsenic	Phosphate mimicry, DNA damage, ROS generation	Cancer, cardiovascular disease, type 2 diabetes

**Table 3:** Trace metals involved in neurodegenerative disease mechanisms.

Mechanism	Related Trace Metals
Neuroinflammation	Zn, Cu, Fe, Mn, Se, Hg, Pb, Cd
Oxidative stress	Zn, Cu, Fe, Mn, Se, Hg, Pb, Cd, As
Neurotransmitter dysfunction	Zn, Cu, Fe, Mn, Hg, Pb, Cd
Beta-amyloid aggregation	Zn, Cu, Fe, Hg
Neuronal damage and apoptosis	Pb, Hg, Cd
Neuronal signaling dysfunction	Pb, Cd, Hg
Intracellular Ca deregulation	Pb, Cd
Mitochondrial dysfunction	Hg, As
Protein misfolding and synaptic dysfunction	Hg
Epigenetic alteration	Pb, Cd
Antioxidant depletion	Hg, Se

**Table 4:** Comparative overview of trace mineral challenges and the role of serum analysis across income-level settings.

Main Problems Involving Trace Minerals	Monitoring Strategies	Priority Applications
Low-income countries		
Deficiencies of Fe, Zn, I, Se; Exposure to As, Cd, Pb	Facilitate detection of hidden hunger and early-stage toxic exposure	Nutritional surveillance Child/maternal health Fortification program design
Middle-income countries		
Coexistence of deficiencies and toxic exposure Dietary transition	Captures dual burden of undernutrition and environmental contamination	Urban vs. rural comparison Monitoring food safety Guiding multisectoral interventions
High-income countries		
Subclinical deficiencies (e.g., Se, Zn) in elderly people or vegans Exposure to Hg, Pb, Cd	Enables personalized assessment of micronutrient imbalances and toxic burden	Geriatric care Personalized nutrition Monitoring of dietary trends

**Table 5:** Biomarkers for essential trace minerals.

	Biomarker	Advantage	Limitation
Cr	Serum/plasma Cr	Useful in exposure and supplementation studies	Reflects recent intake, not long-term status
	Urinary Cr	Effective for monitoring occupational exposure	Insensitive to dietary chromium intake
Cu	Serum Cu	Reflects overall Cu status	Elevated in inflammatory states and pregnancy
	Ceruloplasmin	Indicates functional Cu pool	Acute-phase reactant; influenced by external factors
Fe	Serum ferritin	Reflects Fe stores; widely used in clinical practice	Acute-phase reactant; elevated during inflammation, masking deficiency
	Serum Fe	Indicates circulating Fe levels	Fluctuates due to dietary intake and diurnal rhythms
	Transferrin saturation	Reflects Fe transport capacity	Affected by malnutrition and inflammation
I	Serum thyroglobulin	Reflects I status in thyroid function	Influenced by thyroid disorders; not specific for recent intake
	Urinary I	Gold standard for population studies; reflects recent intake	Does not indicate long-term I status
	Serum TSH	Sensitive indicator of thyroid function; useful in I deficiency disorders	Affected by factors unrelated to I status (e.g., pituitary disorders)
	Serum I	Can reflect recent I intake in individual assessments	High intra-individual variability; not suitable for population-level assessment
Mn	Serum/plasma Mn	Indicates Mn exposure	Rapidly excreted; prone to contamination during sample handling
Mo	Serum/plasma Mo	Reflects short-term intake	No validated biomarkers for deficiency
Se	Serum Se	Reflects recent dietary intake	Not specific to functional Se pool; affected by dietary forms
	Urinary Se	Reflects recent Se intake; useful in population studies	High variability; not reliable for assessing body stores or long-term status
	GPx activity	Reflects functional Se status; used in both plasma and erythrocytes	Sensitive to oxidative stress and inflammation; not specific to Se alone
	SEPP1 activity	Functional marker; reflects Se supply to tissues	Requires advanced assays; not widely available
Zn	Serum/plasma Zn	Simple and widely available	Sensitive to fasting, diurnal variations, and inflammation; low specificity
	Urinary Zn	Reflects recent Zn intake; useful in supplementation studies	Poor correlation with body stores; affected by renal function
	Erythrocyte SOD activity	Reflects functional Zn status; less sensitive to short-term fluctuations	Requires specific assays; less commonly used in clinical settings

**Table 6:** Biomarkers for toxic elements.

	Biomarker	Advantage	Limitation
As	Urinary As	Gold standard for exposure; allows differentiation of toxic forms (inorganic, MMA ^1^, DMA ^2^)	Requires speciation; seafood intake can confound total levels
	Blood As	Reflects very recent exposure	Rapid clearance; less useful for chronic exposure
	Oxidative stress biomarkers (e.g., 8-OHdG)	Reflects As-induced DNA damage	Non-specific; elevated in other conditions
Cd	Urinary Cd	Reflects cumulative body burden, especially renal accumulation; standard for chronic exposure	Affected by kidney function; requires correction for creatinine
	Blood Cd	Indicates recent exposure	Short half-life in blood; not reliable for chronic body load
Pb	Blood Pb	Standard biomarker of recent and chronic exposure	Does not reflect total body burden (especially bone stores)
Hg	Hair Hg	Good indicator of chronic dietary exposure (e.g., fish)	Affected by hair treatment and external contamination
	Blood Hg	Useful for both inorganic and organic forms (with speciation)	Form-specific interpretation needed
	Urinary Hg	Reflects inorganic Hg exposure, particularly occupational exposure	Does not detect methylmercury well

^1^ MMA: monomethylarsonic acid; ^2^ DMA: dimethylarsenic acid; 8-OHdG: 8-hydroxy-2′ deoxyguanosine.

**Table 7:** Levels of essential trace and toxic elements in serum in recent studies. Values are presented as median and lower and upper reference limits (LRL–URL). When these are not available, data are expressed as arithmetic mean (^AM^), geometric mean (^GM^), or range (^R^).

	Australia	Belgium	Denmark	France	Germany	Serbia	Slovenia	Switzerland	USA
	[212]	[213]	[214]	[215]	[216]	[217]	[218]	[219]	[220]
Co									
median	0.47 ^AM^	<LoD	1	0.3	0.12 ^GM^		0.221	0.104	
LRL-URL	0.21–1.3 ^R^	nd–0.8	0.065–0.572		0.04–0.77 ^R^		0.10–1.00	0.059–0.636	
Cu									
median	1100	948	1226	952	1146 ^GM^	729	837	952	1130
LRL-URL	670–2490 ^R^	520–2100	794–2173		560–2280 ^R^	683–774	580–1750	577–1990	
Fe									
median			804						
LRL-URL			313–1675						
I									
median					56 ^GM^			57.6	
LRL-URL					39–118 ^R^			8.68–121.5	
Mn									
median	1	<LoD	1.4	0.65	0.47 ^GM^	1.25	0.493	0.529	
LRL-URL	<1–3.1 ^R^	nd–0.9	0.461–10.37		0.29–0.63 ^R^	1.36–2.09	<LoD-1.00	0.300–1.074	
Mo									
median	0.91			0.61	0.62 ^GM^		0.873	0.878	
LRL-URL	0.26–3 ^R^				0.21–2.7 ^R^		0.45–2.00	0.430–1.886	
Se									
median	130	93.7	110	92	87 ^GM^	65.7	86.8	114.8	127
LRL-URL	82–180 ^R^	65–125	79–150		63–123 ^R^	62.3–70.6	63–120	80.5–162.4	
Zn									
median	1150	762	706	660	903 ^GM^	529	825	784	809
LRL-URL	820–1660 ^R^	480–1150	517–1053		605–1348 ^R^	534–608	600–1450	588–1067	
As									
median			1.21	2.19	0.21 ^GM^		0.172	0.463	
LRL-URL			0.247–12.5		0.02–6.20 ^R^		nd–1.4	<LoD-7.07	
Cd									
median				<0.03	<LoD		<LoD	0.053	
LRL-URL					<0.009–0.033 ^R^		nd–0.50	0.024–0.106	
Cr									
median	<LoD		0.276	<0.29	0.044 ^GM^		<LoD	0.332	
LRL-URL			0.121–0.552		<0.03–0.20 ^R^		nd–0.80	<LoD-1.274	
Hg									
median				0.36	0.12 ^GM^		0.304	0.267	
LRL-URL					<0.02–1.10 ^R^		nd–4.0	0.077–0.993	
Ni									
median		0.62	0.63	0.84	0.28 ^GM^		<LoD	1.085	
LRL-URL		nd–1.2	0.34–1.74		0.17–0.48 ^R^		nd–3.00	<LoD-2.857	
Pb									
median					0.033 ^GM^		<LoD	1.066	
LRL-URL					<0.006–0.13 ^R^		nd–0.85	0.304–4.031	

LoD: limit of detection.
